# Exposure to Workplace Trauma and Posttraumatic Stress Disorder Among Intern Physicians

**DOI:** 10.1001/jamanetworkopen.2021.12837

**Published:** 2021-06-08

**Authors:** Mary C. Vance, Holly B. Herberman Mash, Robert J. Ursano, Zhuo Zhao, Jessica T. Miller, Michael Jeremy D. Clarion, James C. West, Joshua C. Morganstein, Abeer Iqbal, Srijan Sen

**Affiliations:** 1 Center for the Study of Traumatic Stress, Department of Psychiatry, Uniformed Services University, Bethesda, Maryland; 2 Henry M. Jackson Foundation for the Advancement of Military Medicine, Bethesda, Maryland; 3 Michigan Neuroscience Institute, University of Michigan, Ann Arbor; 4 F. Edward Herbert School of Medicine, Uniformed Services University, Bethesda, Maryland

## Abstract

**Importance:**

Physicians are exposed to traumatic events during their work, but the impact and outcomes of these exposures are understudied.

**Objective:**

To determine the prevalence and associations of work-related trauma exposure and posttraumatic stress disorder (PTSD) among a cohort of resident physicians in their internship year of training.

**Design, Setting, and Participants:**

This cohort study involved physicians entering internship at US residency programs nationwide in 2018. Participants completed a baseline survey 1 to 2 months before commencing internship, as well as follow-up surveys at 4 time points during internship. Statistical analysis was performed from April 2020 to January 2021.

**Exposures:**

Twelve months of internship.

**Main Outcomes and Measures:**

Prevalence of work-related trauma and prevalence of PTSD among those who experienced work-related trauma. Trauma exposure and PTSD symptoms were assessed using the Primary Care PTSD Screen for *Diagnostic and Statistical Manual of Mental Disorders* (Fifth Edition) (PC-PTSD-5). Risk factors assessed included depression, anxiety, early family environment, stressful life experiences, medical specialty, hours worked, and concern about medical errors.

**Results:**

Among 1134 interns who completed the PC-PTSD-5 at month 12 of internship, 665 (58.6%) were female and 695 (61.6%) were non-Hispanic White; the mean (SD) age was 27.52 (2.50) years. There were 640 interns (56.4%) who reported work-related trauma exposure; among these interns with trauma exposure, 123 (19.0%) screened positive for PTSD. Overall, 123 of 1134 training physicians (10.8%) screened positive for PTSD by the end of internship year, as compared with a 12-month PTSD prevalence rate of 3.6% in the general population. Multivariable logistic regression analyses, adjusting for demographic characteristics, indicated that risk factors associated with trauma exposure included non-Hispanic White race/ethnicity (odds ratio [OR], 1.51 [95% CI, 1.14-2.01]; *P* = .004), more hours worked (OR, 1.01 [95% CI, 1.00-1.03]; *P* = .03), early family environment (OR, 1.03 [95% CI, 1.01-1.05]; *P* < .001), and stressful life experiences at baseline (OR, 1.46 [95% CI, 1.06-2.01]; *P* = .02). Risk factors associated with PTSD were being unmarried (OR, 2.00 [95% CI, 1.07-3.73]; *P* = .03) and non-Hispanic White (OR, 1.77 [95% CI, 1.01-3.11]; *P* = .05), concern about medical errors (OR, 1.21 [95% CI, 1.00-1.46]; *P* = .05), stressful life experiences during internship (OR, 1.43 [95% CI, 1.14-1.81]; *P* = .002), depression at month 12 of internship (OR, 2.52 [95% CI = 1.36-4.65], *P* = .003), and anxiety at month 12 of internship (OR, 2.14, [95% CI, 1.13-4.04]; *P* = .02).

**Conclusions and Relevance:**

This study found that work-related PTSD was 3 times more prevalent among intern physicians than the general population. These findings suggest that effective interventions to reduce trauma exposure and mitigate the effects of trauma are needed.

## Key Points

**Question:**

What is the prevalence of work-related trauma exposure, and what factors are associated with posttraumatic stress disorder among intern physicians?

**Findings:**

In this cohort study of 1134 interns, 56.4% reported trauma exposure during internship, and 19.0% of those experiencing trauma screened positive for posttraumatic stress disorder. Risk factors for trauma exposure included non-Hispanic White race/ethnicity, more hours worked, early family environment, and baseline stressful life experiences; risk factors for posttraumatic stress disorder included being unmarried and non-Hispanic White, concern about medical errors, stressful life experiences during internship, and depression or anxiety at 12 months.

**Meaning:**

This study’s findings suggest that work-related trauma exposure and posttraumatic stress disorder are prevalent among interns, and interventions may improve physician well-being.

## Teaser

This cohort study examines the prevalence and associations of work-related trauma exposure and posttraumatic stress disorder among physician interns in US residency programs.

## Introduction

Posttraumatic stress disorder (PTSD) is a potentially debilitating condition characterized by intrusive memories, avoidance, negative alterations in mood and cognition, and hyperarousal after exposure to a traumatic stressor. An estimated 70.4% of the adult population worldwide experiences a traumatic event in their lifetime.^1^ Of those, approximately 6.8% develop PTSD over their lifetime,^2^ with a prevalence rate of 3.6% over the past year.^3^ Changes in the *Diagnostic and Statistical Manual of Mental Disorders* (Fifth Edition) (*DSM-5*) diagnostic criteria for PTSD^4^ indicate that work-related traumatic exposures are PTSD-qualifying events.^5^

Physicians are at risk for work-related exposure to traumatic events, including patient critical illness and death, serious medical errors and complications, treating people exposed to natural and human-generated disasters, workplace violence, and hazardous exposures (eg, the COVID-19 pandemic).^6^ Risk for traumatic exposure is particularly pronounced for resident physicians, who typically have the greatest responsibility for frontline clinical care with high patient volumes, and lack the training and experience of more seasoned physicians. However, in contrast to the burgeoning literature on burnout,^7^ depression,^8^ and suicide,^9^ little is known about the prevalence and associations of trauma exposure and PTSD in physicians and physicians-in-training.

Risk of work-related trauma exposure and PTSD among health care professionals is increasingly recognized, especially since the beginning of the COVID-19 pandemic.^10^ Despite this, most prior research on PTSD in physicians consists of small studies of specific medical specialties at a limited number of institutions. Of the 3 studies we identified with more than 1000 participants, all found considerable rates of PTSD symptoms or probable PTSD in physician populations.^11,12,13^ However, there have been no nationwide longitudinal studies of work-related trauma exposure and PTSD among physicians-in-training—a time when trauma exposure and the resultant mental health consequences may substantially impact the ability to learn and care for patients. This is a costly gap in our knowledge of the determinants of physician well-being, because it has been demonstrated that mental distress and illness in physicians contributes to poor quality of care and increased care costs and medical errors.^14,15^ The current study seeks to address that knowledge gap by longitudinally assessing the prevalence and associations of work-related trauma exposure and PTSD in a well-characterized, nationwide sample of US intern physicians (in their first year of residency training).

## Methods

### Participants

Individuals entering residency at collaborating institutions during the 2018-2019 academic year were invited to participate via email 2 months before starting internship. In total, 4350 invitation emails were sent to interns entering residency programs in internal medicine, surgery, obstetrics/gynecology, pediatrics, psychiatry, emergency medicine, combined medicine and pediatrics, family practice, anesthesiology, transitional, and other specialties (eg, child neurology, orthopedic surgery, preliminary years, and other specialties with samples too small to list individually). For 14 individuals, emails were returned as undeliverable and we were unable to obtain a valid email address. Of the remaining invited individuals, 2129 of 4350 (49%) agreed to participate. The institutional review board at the University of Michigan and participating hospitals approved the larger Intern Health Study, and the Uniformed Services University institutional review board determined that data analysis for the current study was exempt because it is not human subjects research. Participants provided electronic informed consent and were given $125 gift certificates as compensation ($25 per survey completed). This study followed the Strengthening the Reporting of Observational Studies in Epidemiology (STROBE) reporting guideline for cohort studies.

### Data Collection

Participants completed a baseline survey 1 to 2 months before commencing internship. The survey assessed demographic characteristics (age, sex, race/ethnicity, marital status), medical education factors (medical specialty, training institution), psychological factors (self-reported history of depression), and psychosocial factors (sexual orientation, early family environment, and stressful life events over the past 3 months). Racial and ethnic groups other than non-Hispanic White were collapsed into a single category because of small sample size.

Participants were contacted again via email at months 3, 6, 9, and 12 of their internship year and asked to complete follow-up surveys. At each time point, interns were asked about (1) stressful life events and concern over perceived medical errors in the past 3 months and (2) number of hours worked over the past week. At month 12, current PTSD, depression, and anxiety symptoms were assessed.

### Measures

Trauma exposure and PTSD symptoms were assessed using the Primary Care PTSD Screen for *DSM-5* (PC-PTSD-5), a 5-item screening questionnaire for probable or possible PTSD, reflecting *DSM-5* PTSD diagnostic criteria.^16^ The PC-PTSD-5 is diagnostically accurate, with content and face validity relative to the *DSM-5*.^16^ The PC-PTSD-5 begins with a screen for trauma exposure, which we adapted for this study to specify health care–specific traumatic exposures: “Sometimes things happen to physicians that are unusually or especially frightening, horrible, or traumatic. For example: sudden patient deaths, serious medical errors, workplace violence, hazardous exposure, or repeated or extreme exposure to the details of traumatic events. Have you ever experienced this kind of event as a physician?” Respondents with no reported trauma exposure were recorded with a negative screen, whereas those who responded yes to the first item completed 5 questions assessing PTSD symptoms over the past month (with yes or no response options) for a total score of 0 to 5. A cutoff score of 3 established a possible PTSD diagnosis in this study, which has been demonstrated to be maximally sensitive for the detection of PTSD.^16^

Depressive symptoms within the past 2 weeks were assessed using the 9-item Patient Health Questionnaire (PHQ-9), a commonly used and validated self-report inventory.^17^ Each item yields a score ranging from 0 (not at all) to 3 (nearly every day), with a total score range of 0 to 27. A score of 10 or greater has been found to indicate moderate to severe depression, with a sensitivity of 93% and a specificity of 88%.^18^ Self-reported history of depression was assessed using a single yes or no item: “To the best of your recollection, have you EVER experienced an episode of depression (a two week period of your life when you felt down or lost interest or pleasure in your usual activities and also had difficulty concentrating or noticed changes in sleep, appetite, energy or experienced thoughts of death or feelings of guilt)?”

Symptoms of generalized anxiety disorder (GAD) over the past 2 weeks were measured using the Generalized Anxiety Disorder 7-item scale (GAD-7), which has demonstrated reliability and validity.^19^ Item responses range from 0 (not at all) to 3 (nearly every day), with a possible total score range of 0 to 21. A cutoff point of 10 has been determined to correspond with a moderate level of GAD, with a sensitivity of 89% and a specificity of 82%.

Early family environment was assessed using the Risky Families Questionnaire (RFQ), a validated 13-item retrospective self-report measure assessing abuse, neglect, and family conflict between ages 5 to 15 years.^20^ Each item is rated on a 5-point scale ranging from 1 (not at all) to 5 (very often), with higher scores indicating more exposure to risky experiences and conflict during childhood. Recent stressful life events over the past 3 months were assessed using an 11-item scale developed for this study.^21^ Participants responded yes or no to items addressing several stressful event categories, including loss of a family member, injury or illness, financial difficulties, changes in relationship and parental status, and exposure to physical violence, yielding a score of 1 at each time point if participants responded yes to any item (eFigure in the Supplement). Concern that participants made a major medical error in the last 3 months was assessed as yes or no. Cumulative scores for quarter 1 to quarter 4 responses to the stressful life events and concern about medical errors items were calculated, with possible ranges 0 to 4.

### Statistical Analysis

We used descriptive statistics to calculate prevalence of work-related trauma exposure in our sample and prevalence of PTSD among those with work-related trauma exposure. We conducted 2 sets of univariable and multivariable logistic regression models to examine associations of demographic, psychological, psychosocial, and work-related factors with (1) work-related trauma exposure and (2) PTSD after work-related trauma exposure.

#### Association of Covariates With Trauma Exposure

First, we conducted a series of separate univariable logistic regression analyses to examine associations of each of the demographic characteristics with work-related trauma exposure, and then examined all of the demographic characteristics together in a multivariable model testing associations with trauma exposure. We subsequently performed a series of separate multivariable logistic regression analyses, adjusting for demographic characteristics, to separately examine each psychological, psychosocial, and work-related factor’s association with trauma exposure. Psychological, psychosocial, and work-related factors found to be significantly associated with trauma exposure in separate analyses were then entered together into a final multivariable model, again controlling for demographic characteristics.

#### Association of Covariates With PTSD

The aforementioned steps were repeated in a separate set of univariable and multivariable logistic regression models to assess the associations between these factors and PTSD following work-related trauma exposure. Logistic regression coefficients were exponentiated to obtain odds ratios (ORs) and 95% CIs. The significance threshold was *P* ≤ .05. We used Wald χ^2^ to calculate significance, and testing was 2-sided. Statistical analyses were conducted using SPSS Statistics version 25.0 (IBM Corp)^22^ from April 2020 to January 2021. Listwise deletion was used to account for missing data.

## Results

Our analytic sample included 1134 participants who responded to the PC-PTSD-5 work-related trauma exposure item at 12 months. Among these participants, 665 (58.6%) were female, 695 (61.6%) were non-Hispanic White, 823 (72.6%) were unmarried, 574 (50.6%) lived with a significant other, 1054 (92.9%) had no children, and 1023 (90.8%) identified as heterosexual (vs lesbian, gay, bisexual, transgender, questioning); the mean (SD) age of participants was 27.52 (2.50) years (Table 1). Our overall response rate was 26% (1134 respondents [at 12 months] of 4350 total invitees [at baseline]), with response rates differing by specialty at 12 months: 19% (225 of 1159) of surgical interns invited to participate at baseline responded at 12 months, and 28% (907 of 3191) of nonsurgical interns invited to participate at baseline responded at 12 months.

**Table 1.** Demographic and Work-Related Characteristics Among the Trauma Exposure and PTSD Groups

Characteristic	No. (%)				
	Trauma exposure^a^		PTSD^b^		Total sample
	No	Yes	No	Yes	
No.	494	640	517	123	1134
Sex					
Male	207 (41.9)	262 (40.9)	221 (42.7)	41 (33.3)	469 (41.4)
Female	287 (58.1)	378 (59.1)	296 (57.3)	82 (66.7)	665 (58.6)
Race/ethnicity					
non-Hispanic White	281 (57.3)	414 (64.8)	327 (63.4)	87 (70.7)	695 (61.6)
Non-White^c^	209 (42.7)	225 (35.2)	189 (36.6)	36 (29.3)	434 (38.4)
Marital status					
Not married	358 (72.5)	465 (72.7)	368 (71.2)	97 (78.9)	823 (72.6)
Married	136 (27.5)	175 (27.3)	149 (28.8)	26 (21.1)	311 (27.4)
Live with significant other					
No	250 (50.6)	310 (48.4)	246 (47.6)	64 (52.0)	560 (49.4)
Yes	244 (49.4)	330 (51.6)	271 (52.4)	59 (48.0)	574 (50.6)
Have children					
No	451 (91.3)	603 (94.2)	486 (94.0)	117 (94.2)	1054 (92.9)
Yes	43 (8.7)	37 (5.8)	31 (6.0)	6 (4.9)	80 (7.1)
Age, mean (SD) [range]	27.53 (2.62) [23-41]	27.51 (2.40) [23-44]	27.53 (2.42) [23-44]	27.45 (2.33) [23-37]	27.52 (2.50) [23-44]
Sexual orientation					
Heterosexual	455 (92.5)	568 (89.4)	464 (90.3)	104 (86.0)	1023 (90.8)
LGBTQ	37 (7.5)	67 (10.6)	50 (9.7)	17 (14.0)	104 (9.2)
Total	494 (43.6)	640 (56.4)	517 (80.8)	123 (19.2)	1134 (100)

Abbreviations: LGBTQ, lesbian, gay, bisexual, transgender, queer/questioning; PC-PTSD-5, Primary Care PTSD Screen for *Diagnostic and Statistical Manual of Mental Disorders* (Fifth Edition); PTSD, posttraumatic stress disorder.

^a^ Intern physician report of being exposed to a work-related traumatic event during the past year (0 = no, 1 = yes).

^b^ Possible PTSD is defined as scoring 3 or greater on the PC-PTSD-5 during quarter 4 (0 = no, 1 = yes).

^C^ These races/ethnicities included African American, Latino, Asian, Native American, Arab/Middle Eastern, mixed, and other.

Of our 1134 respondents, 640 (56.4%) reported work-related trauma exposure, and all answered the PC-PTSD-5 screening questions. Among the 640 individuals who reported work-related trauma exposure, 123 (19.0%) screened positive for PTSD. The overall rate of possible PTSD among interns in the study sample was 10.8% (123 of 1134).

### Work-Related Trauma Exposure

Trauma exposure across specialties ranged from 43.1% (anesthesiology: 25 of 58 respondents) to 72.4% (emergency medicine: 71 of 98 respondents) (Table 2). In our logistic regression model assessing for associations with trauma exposure, we found that only race/ethnicity was associated with trauma exposure, with non-Hispanic White interns significantly more likely to report trauma exposure (OR, 1.37 [95% CI, 1.08-1.74]; *P* = .01) (eTable 1 in the Supplement). In a multivariable model including all of the demographic characteristics, this association of non-Hispanic White race/ethnicity with trauma exposure remained significant (OR, 1.37 [95% CI, 1.07-1.75]; *P* = .01) (eTable 1 in the Supplement).

**Table 2.** Trauma Exposure and PTSD Prevalence Rates by Medical Specialty

Medical Specialty	Participants, No. (%)				
	Trauma exposure^a^		PTSD^b^		Total^c^
	No	Yes	No	Yes	
Internal medicine	106 (43.4)	138 (56.6)	105 (76.1)	33 (23.9)	244 (21.5)
Surgery	33 (36.7)	57 (63.3)	50 (87.7)	7 (12.3)	90 (8.0)
Obstetrics/gynecology	37 (48.1)	40 (51.9)	37 (92.5)	3 (7.5)	77 (6.8)
Pediatrics	76 (45.8)	90 (54.2)	63 (70)	27 (30.0)	166 (14.7)
Psychiatry	26 (40.6)	38 (59.4)	35 (92.1)	3 (7.9)	64 (5.7)
Emergency medicine	27 (27.6)	71 (72.4)	58 (81.7)	13 (18.3)	98 (8.7)
Medicine/pediatrics	9 (29.0)	22 (71.0)	16 (72.7)	6 (27.3)	31 (2.7)
Family practice	51 (48.6)	54 (51.4)	40 (74.1)	14 (25.9)	105 (9.3)
Other	70 (45.5)	84 (54.5)	74 (88.1)	10 (11.9)	154 (13.6)
Transitional	24 (53.3)	21 (46.7)	17 (81.0)	4 (19.0)	45 (4.0)
Anesthesiology	33 (56.9)	25 (43.1)	22 (88.0)	3 (12.0)	58 (5.1)

Abbreviations: PC-PTSD-5, Primary Care PTSD Screen for *Diagnostic and Statistical Manual of Mental Disorders* (Fifth Edition); PTSD, posttraumatic stress disorder.

^a^ Intern physician report of being exposed to a work-related traumatic event during the past year (0 = no, 1 = yes). Percentage indicated is among total number of participants in each specialty who were or were not exposed to a work-related trauma.

^b^ Possible PTSD is defined as scoring 3 or greater on the PC-PTSD-5 during Q4 (0 = no, 1 = yes). Percentage indicated is among participants in each specialty who were exposed to a work-related trauma and did or did not have possible PTSD.

^c^ Total No. (%) refer to the participants in each specialty out of the total N of 1134 participants.

When entered separately into models controlling for demographic characteristics, the following factors were significantly associated with trauma exposure: specialty (combined medicine and pediatrics: OR, 3.15 [95% CI, 1.58-6.30]; *P* = .001; family practice: OR, 2.90 [95% CI, 1.13-7.44]; *P* = .03; obstetrics/gynecology: OR, 2.07 [95% CI, 1.05-4.09]; *P* = .04, with each specialty more likely to be associated with trauma exposure than internal medicine); mean number of hours worked (OR, 1.01 [95% CI, 1.00-1.02], *P* = .01); early family environment (OR, 1.03 [95% CI, 1.02-1.05]; *P* < .001); stressful life experiences at baseline (OR, 1.70 [95% CI, 1.28-2.25]; *P* < .001); stressful life experiences during internship (OR, 1.16 [95% CI, 1.03-1.33]; *P* = .02); lifetime history of depression (OR, 1.47 [95% CI, 1.16-1.84]; *P* = .002); and current depression at month 12 (OR, 1.58 [95% CI, 1.17-2.14]; *P* = .003) (eTable 2 in the Supplement).

Factors significant in separate models were then entered together into a final model, controlling for demographic characteristics. In this model, non-Hispanic White race/ethnicity (OR, 1.51 [95% CI, 1.14-2.01]; *P* = .004), more hours worked (OR, 1.01 [95% CI, 1.00-1.03]; *P* = .03), early family environment (OR = 1.03 [95% CI, 1.01-1.05]; *P* < .001]), and stressful life experiences at baseline (OR, 1.46 [95% CI, 1.06-2.01]; *P* = .02) remained associated with trauma exposure (Table 3).

**Table 3.** Multivariable Associations of Work-Related, Psychosocial, and Psychological Factors With Work-Related Trauma Exposure^a^

Characteristic	OR (95% CI)
Work-related factors	
Specialty	
Internal medicine	1 [Reference]
Surgery	1.77 (0.93-3.35)
Obstetrics/gynecology	1.76 (0.82-3.79)
Pediatrics	1.41 (0.65-3.09)
Psychiatry	1.65 (0.84-3.25)
Emergency medicine	1.86 (0.82-4.20)
Medicine/pediatrics	3.25 (1.53-6.92)
Family practice	2.90 (1.07-7.87)
Transitional	1.42 (0.68-2.97)
Anesthesiology	1.61 (0.81-3.17)
Other	1.53 (0.64-3.67)
χ^2^_10_	13.39
Mean No. of hours worked^b^	1.01 (1.00-1.03)
χ^2^_1_	4.55
Psychosocial factors	
Early family environment	1.03 (1.01-1.05)
χ^2^_1_	12.78
Stressful life experiences (baseline)^c^	1.46 (1.06-2.01)
χ^2^_1_	5.50
Stressful life experiences (Q1 to Q4)^d^	1.11 (0.97-1.27)
χ^2^_1_	2.19
Psychological factors	
Lifetime history of depression^e^	0.84 (0.64-1.11)
χ^2^_1_	1.47
Current depression (Q4)^f^	1.17 (0.82-1.66)
χ^2^_1_	0.73

Abbreviations: OR, odds ratio; PHQ-9, Patient Health Questionnaire-9; Q, quarter.

^a^ Variables that were significant when entered in a series of separate multivariable models that adjusted for demographic characteristics (sex, current age, race/ethnicity, and marital status) (eTable 2 in the Supplement) were entered together in a final multivariable model, adjusting for demographics.

^b^ Mean of hours reported (Q1 to Q4).

^c^ Cumulative stressful life experiences (0 = no, 1 = yes; baseline = past 3 months).

^d^ Cumulative stressful life experiences (0 = no, 1 = yes; Q1 to Q4).

^e^ Lifetime history of depression reported at baseline.

^f^ Current depression: scoring 10 or higher on the PHQ-9, indicating moderate to severe depression (0 = no, 1 = yes), assessing symptoms over the past 2 weeks.

### Work-Related PTSD

Among those with trauma exposure, prevalence rates of possible PTSD across specialties ranged from 7.5% (obstetrics/gynecology: 3 of 40 respondents) to 30.0% (pediatrics: 27 of 90 respondents) (Table 2). In univariable logistic regression analyses, no demographic characteristics were significantly associated with PTSD (eTable 3 in Supplement). However, in multivariable analyses that included all demographic characteristics, unmarried status was significantly associated with PTSD (OR, 1.65 [95% CI, 1.01-2.69]; *P* = .05) (eTable 3 in the Supplement).

When entered separately into multivariable logistic regression models controlling for demographic characteristics, the following work-related, psychosocial, and psychological factors were significantly associated with PTSD: specialty (surgery: OR, 0.40 [95% CI, 0.16-0.97]; *P* = .04; obstetrics/gynecology: OR, 0.22 [95% CI, 0.06-0.78]; *P* = .03; psychiatry: OR = 0.27 [95% CI, 0.08-0.93]; *P* = .04; other specialties: OR, 0.39 [95% CI, 0.17-0.86]; *P* = .02, with interns in these specialties less likely to report PTSD than those in internal medicine), concern about medical errors (OR, 1.36 [95% CI, 1.15-1.60]; *P* < .001), stressful life experiences during internship (OR, 1.56 [95% CI, 1.27-1.91], *P* < .001), lifetime history of depression (OR, 2.29 [95% CI, 1.50-3.51]; *P* < .001), current depression at month 12 (OR, 3.96 [95% CI, 2.58-6.08]; *P* < .001), and current anxiety at month 12 (OR, 4.41 [95% CI, 2.81-6.92]; *P* < .001) (eTable 4 in Supplement).

When all of these significant work-related, psychosocial, and psychological factors were entered into a final model controlling for demographic characteristics, all factors remained significant except for lifetime history of depression, with the strongest positive association found between current depression and PTSD (OR, 2.52 [95% CI, 1.36-4.65]; *P* < .001) (Table 4). Unmarried status and non-Hispanic White race/ethnicity were also significantly associated with PTSD in the final model (OR, 2.00 [95% CI, 1.07-3.73]; *P* = .03; and OR, 1.77 [95% CI, 1.01-3.11]; *P* = .05, respectively).

**Table 4.** Multivariable Associations of Work-Related, Psychosocial, and Psychological Factors With Work-Related PTSD^a^^,^^b^

Characteristic	OR (95% CI)
Work-related factors	
Specialty	
Internal medicine	1 [Reference]
Surgery	0.26 (0.09-0.81)
Obstetrics/gynecology	0.14 (0.03-0.66)
Pediatrics	1.23 (0.57-2.66)
Psychiatry	0.15 (0.03-0.77)
Emergency medicine	0.49 (0.19-1.21)
Medicine/pediatrics	1.30 (0.41-4.05)
Family practice	1.07 (0.44-2.58)
Transitional	0.52 (0.13-2.15)
Anesthesiology	0.48 (0.12-1.95)
Other	0.32 (0.12-0.85)
χ^2^_10_	24.06
Concern about medical errors^c^	1.21 (1.00-1.46)
χ^2^_1_	3.84
Psychosocial factors	
Stressful life experiences (Q1 to Q4)^d^	1.43 (1.14-1.81)
χ^2^_1_	9.21
Psychological factors	
Lifetime history of depression	1.70 (0.99-2.90)
χ^2^_1_	3.73
Current depression (Q4)^e^	2.52 (1.36-4.65)
χ^2^_1_	8.65
Current anxiety (Q4)^f^	2.14 (1.13-4.04)
χ^2^_1_	5.48

Abbreviations: PTSD, posttraumatic stress disorder; OR, odds ratio; Q, quarter.

^a^ Variables that were significant when entered in a series of separate multivariable models that adjusted for demographics (sex, current age, race/ethnicity, and marital status) (eTable 4 in the Supplement) were entered together in a final multivariable model, adjusting for demographics.

^b^ Possible PTSD is defined as scoring 3 or greater on the PC-PTSD-5 during Q4.

^c^ Cumulative concern about major medical errors (0 = no, 1 = yes; Q1 to Q4).

^d^ Cumulative stressful life experiences (0 = no, 1 = yes; Q1 to Q4).

^e^ Current depression: scoring 10 or higher on the PHQ-9, indicating moderate to severe depression (0 = no, 1 = yes).

^f^ Current anxiety: Scoring 10 or higher on the GAD-7 (0 = no, 1 = yes).

## Discussion

In this well-characterized, longitudinal cohort of 1134 training physicians in multiple specialties across institutions nationwide, we found that more than half of interns (56.4%) reported work-related trauma exposure, and approximately1 in 5 of those with trauma exposure screened positive for possible PTSD (19.0%). Approximately 1 in 10 training physicians (10.8%) screened positive for possible PTSD by the end of internship year. As compared with the estimated 12-month PTSD prevalence rate of 3.6% in the general population,^3^ our 12-month PTSD rates in training physicians were 3-fold higher. These rates of possible PTSD in physicians align with those previously found in the literature, which range from approximately 14% to 25%,^11,12,13^ and underscore the need to recognize and intervene on this mental health problem.

Different risk and protective factors were associated with work-related trauma exposure vs PTSD. Most of the risk factors for trauma exposure were non–work-related, static risk factors established before commencement of internship, such as non-Hispanic White race/ethnicity, early family environment, and stressful life experiences at baseline. A possible explanation for the associations of early family environment and previous stressful events with trauma exposure is that a priming effect of prior stressful or traumatic life events (eg, abusive growing-up experience, death of a loved one) could predispose individuals to subsequently experience or perceive additional traumatic exposures—a phenomenon known as revictimization.^23^ Higher number of hours worked was the only work-related factor significantly associated with trauma exposure. It may be that working longer hours generates a higher burden of stress and presents a longer time window for exposure to work-related traumatic events.

In contrast to the static, preinternship factors that were largely associated with work-related trauma exposure, factors associated with PTSD were more likely to be work-related (specialty, concern about medical errors) or occur during internship (stressful life experiences during internship, current depression or anxiety at month 12). Many of these associations are clinically intuitive: adverse events such as medical errors can precipitate psychological distress in health care workers,^24^ and stress and mental illness during internship may predispose individuals to other mental health comorbidities.

Of note, non-Hispanic White race/ethnicity was found to be associated with both trauma exposure and PTSD. The reasons for this are unclear, and this link should be interpreted with caution. Our findings contrast with the existing literature, which suggests that, although non-Hispanic White interns reported higher rates of trauma exposure, certain racial/ethnic minorities have a higher prevalence of PTSD even after adjusting for type and frequency of trauma exposure.^25,26^ Moreover, experts have cautioned against clinical and policy decision-making based on race, even when such findings are supported by empirical data.^27^

The associations between specific medical specialties and risk of work-related trauma exposure and PTSD also warrant further discussion. In our model on trauma exposure, there was no significant association overall between medical specialty and trauma exposure. However, in our model on PTSD, 2 specialties were found to be less associated with PTSD following work-related trauma exposure: surgery and psychiatry (specialties categorized as other were also protective in our analysis, but this category is too heterogeneous to remark upon). These findings, however, should be placed into context: as we used internal medicine (the most common specialty in our sample) as the reference category, it does not provide information on differences between specialties other than internal medicine. Therefore, examining effect sizes for trauma exposure and PTSD between specialties (Table 3 and Table 4, respectively), as well as prevalence rates of trauma exposure and PTSD within each specialty (Table 2), is likely to yield more practically meaningful information than relying on statistical significance alone.

### Limitations

This study should be considered in light of its limitations. Self-report data are subject to recall bias. Our response rate was relatively low, at 26% overall (19% for surgical specialties and 28% for nonsurgical specialties). The lower response rate among surgical interns may be due to their more intensive work schedule, resulting in less time to participate, which may have introduced self-selection bias and limited generalizability to all US intern physicians. Attrition was managed by maintaining regular contact with participants.

The PC-PTSD-5 is a screening questionnaire, and as such cannot be interpreted as being diagnostic for PTSD. As PTSD symptoms were only assessed at month 12, we cannot ascertain whether participants developed PTSD during internship or already had PTSD at baseline. However, this concern is mitigated by the fact that the PC-PTSD-5 asked specifically about trauma exposure “as a physician” (ie, work-related trauma exposure) and elicited PTSD symptoms specifically associated with work-related traumatic events, thereby increasing the likelihood that participants reported PTSD symptoms associated with their work vs life events occurring before internship. Because the PC-PTSD-5 is a self-report instrument, it may not collect information on trauma exposure and PTSD symptoms as reliably as a clinician-administered instrument. Although our study evaluated a wide range of risk and protective factors for trauma exposure and PTSD, it is not comprehensive. Future studies should also examine additional factors previously found to be associated with physician mental health (eg, work-life balance,^12,13^ workplace depersonalization or bullying,^12,13^ perceived support following trauma exposure).^28,29,30^

## Conclusions

This study provides additional support to the existing literature on trauma exposure and PTSD among physicians by longitudinally assessing work-related trauma exposure and PTSD among intern physicians in a large, well-characterized nationwide sample. More research is needed to determine the prevalence of trauma exposure and PTSD at different stages of a physician’s career. Also needed are studies on specific groups of physicians and physicians in training who may be at particularly high risk for work-related trauma exposure and PTSD (eg, in medical specialties with high rates of trauma exposure or PTSD, from racial/ethnic groups who may develop PTSD at higher rates). Finally, studies to identify effective interventions following work-related trauma exposure in physicians are needed, with the goal of enhancing physician performance, increasing professional satisfaction, and ultimately improving patient care.

## Notes

Supplement. **eTable 1.** Association of Demographic Characteristics of Intern Physicians With Work-Related Trauma Exposure **eTable 2.** Multivariable Associations of Work-Related, Psychosocial, and Psychological Factors With Work-Related Trauma Exposure **eTable 3.** Association of Demographic Characteristics of Intern Physicians With Work-Related PTSD **eTable 4.** Multivariable Associations of Work-Related, Psychosocial, and Psychological Factors With Work-Related PTSD **eFigure.** Questionnaire Assessing Stressful Life Events Over the Past Three Months

## References

[1] Kessler RC, Aguilar-Gaxiola S, Alonso J, . Trauma and PTSD in the WHO world mental health surveys. Eur J Psychotraumatol. 2017;8(sup5):1353383. doi:10.1080/20008198.2017.1353383 29075426 PMC5632781

[2] Harvard Medical School. National Comorbidity Survey (NCS). Table 1: lifetime prevalence DSM-IV/WMH-CIDI disorders by sex and cohort. Published 2007. Accessed April 28, 2021. https://www.hcp.med.harvard.edu/ncs/ftpdir/NCS-R_Lifetime_Prevalence_Estimates.pdf

[3] Harvard Medical School. National Comorbidity Survey (NCS). Table 2: 12-month prevalence DSM-IV/WMH-CIDI disorders by sex and cohort. Published 2007. Accessed April 28, 2021. https://www.hcp.med.harvard.edu/ncs/ftpdir/NCS-R_12-month_Prevalence_Estimates.pdf

[4] American Psychiatric Association. Diagnostic and Statistical Manual of Mental Disorders. American Psychiatric Publishing; 2013.

[5] Friedman MJ, Resick PA, Bryant RA, Brewin CR. Considering PTSD for DSM-5. Depress Anxiety. 2011;28(9):750-769. doi:10.1002/da.20767 21910184

[6] Morganstein JC, West JC, Ursano RJ. Work-associated trauma. In:Brower KJ, Riba MB, eds. Physician Mental Health and Well-Being. Springer; 2017:33-60.

[7] Rotenstein LS, Torre M, Ramos MA, . Prevalence of burnout among physicians: a systematic review. JAMA. 2018;320(11):1131-1150. doi:10.1001/jama.2018.12777 30326495 PMC6233645

[8] Mata DA, Ramos MA, Bansal N, . Prevalence of depression and depressive symptoms among resident physicians: a systematic review and meta-analysis. JAMA. 2015;314(22):2373-2383. doi:10.1001/jama.2015.15845 26647259 PMC4866499

[9] Duarte D, El-Hagrassy MM, Couto TCE, Gurgel W, Fregni F, Correa H. Male and female physician suicidality: a systematic review and meta-analysis. JAMA Psychiatry. 2020;77(6):587-597. doi:10.1001/jamapsychiatry.2020.0011 32129813 PMC7057173

[10] Carmassi C, Foghi C, Dell’Oste V, . PTSD symptoms in healthcare workers facing the three coronavirus outbreaks: what can we expect after the COVID-19 pandemic. Psychiatry Res. 2020;292:113312. doi:10.1016/j.psychres.2020.113312 32717711 PMC7370915

[11] Harrison R, Lawton R, Stewart K. Doctors’ experiences of adverse events in secondary care: the professional and personal impact. Clin Med (Lond). 2014;14(6):585-590. doi:10.7861/clinmedicine.14-6-585 25468840 PMC4954127

[12] Jackson T, Morgan J, Jackson D, . Trends in surgeon wellness (take a sad song and make it better): a comparison of surgical residents, fellows, and attendings. Am Surg. 2019;85(6):579-586. doi:10.1177/000313481908500620 31267897

[13] Jackson T, Zhou C, Khorgami Z, . Traumatized residents - it’s not surgery. it’s medicine. J Surg Educ. 2019;76(6):e30-e40. doi:10.1016/j.jsurg.2019.08.002 31477549

[14] West CP, Dyrbye LN, Shanafelt TD. Physician burnout: contributors, consequences and solutions. J Intern Med. 2018;283(6):516-529. doi:10.1111/joim.12752 29505159

[15] Fahrenkopf AM, Sectish TC, Barger LK, . Rates of medication errors among depressed and burnt out residents: prospective cohort study. BMJ. 2008;336(7642):488-491. doi:10.1136/bmj.39469.763218.BE 18258931 PMC2258399

[16] Prins A, Bovin MJ, Smolenski DJ, . The primary care PTSD screen for DSM-5 (PC-PTSD-5): development and evaluation within a veteran primary care sample. J Gen Intern Med. 2016;31(10):1206-1211. doi:10.1007/s11606-016-3703-5 27170304 PMC5023594

[17] Spitzer RL, Kroenke K, Williams JB; Primary Care Evaluation of Mental Disorders. Validation and utility of a self-report version of PRIME-MD: the PHQ primary care study. primary care evaluation of mental disorders. patient health questionnaire. JAMA. 1999;282(18):1737-1744. doi:10.1001/jama.282.18.1737 10568646

[18] Kroenke K, Spitzer RL, Williams JB. The PHQ-9: validity of a brief depression severity measure. J Gen Intern Med. 2001;16(9):606-613. doi:10.1046/j.1525-1497.2001.016009606.x 11556941 PMC1495268

[19] Spitzer RL, Kroenke K, Williams JB, Löwe B. A brief measure for assessing generalized anxiety disorder: the GAD-7. Arch Intern Med. 2006;166(10):1092-1097. doi:10.1001/archinte.166.10.1092 16717171

[20] Taylor SE, Lerner JS, Sage RM, Lehman BJ, Seeman TE. Early environment, emotions, responses to stress, and health. J Pers. 2004;72(6):1365-1393. doi:10.1111/j.1467-6494.2004.00300.x 15509286

[21] Sen S, Kranzler HR, Krystal JH, . A prospective cohort study investigating factors associated with depression during medical internship. Arch Gen Psychiatry. 2010;67(6):557-565. doi:10.1001/archgenpsychiatry.2010.41 20368500 PMC4036806

[22] IBM. SPSS Statistics. Accessed April 23, 2021. https://www.ibm.com/products/spss-statistics

[23] Jaffe AE, DiLillo D, Gratz KL, Messman-Moore TL. Risk for revictimization following interpersonal and noninterpersonal trauma: clarifying the role of posttraumatic stress symptoms and trauma-related cognitions. J Trauma Stress. 2019;32(1):42-55. doi:10.1002/jts.22372 30748027 PMC6599470

[24] Seys D, Wu AW, Van Gerven E, . Health care professionals as second victims after adverse events: a systematic review. Eval Health Prof. 2013;36(2):135-162. doi:10.1177/0163278712458918 22976126

[25] Roberts AL, Gilman SE, Breslau J, Breslau N, Koenen KC. Race/ethnic differences in exposure to traumatic events, development of post-traumatic stress disorder, and treatment-seeking for post-traumatic stress disorder in the United States. Psychol Med. 2011;41(1):71-83. doi:10.1017/S0033291710000401 20346193 PMC3097040

[26] Alegría M, Fortuna LR, Lin JY, . Prevalence, risk, and correlates of posttraumatic stress disorder across ethnic and racial minority groups in the United States. Med Care. 2013;51(12):1114-1123. doi:10.1097/MLR.0000000000000007 24226308 PMC3922129

[27] Vyas DA, Eisenstein LG, Jones DS. Hidden in plain sight - reconsidering the use of race correction in clinical algorithms. N Engl J Med. 2020;383(9):874-882. doi:10.1056/NEJMms2004740 32853499

[28] van Steijn ME, Scheepstra KWF, Yasar G, Olff M, de Vries MC, van Pampus MG. Occupational well-being in pediatricians-a survey about work-related posttraumatic stress, depression, and anxiety. Eur J Pediatr. 2019;178(5):681-693. doi:10.1007/s00431-019-03334-7 30783762 PMC6459799

[29] Carter F, Bell C, Ali A, . Predictors of psychological resilience amongst medical students following major earthquakes. N Z Med J. 2016;129(1434):17-22. 27349259

[30] Klamen DL, Grossman LS, Kopacz D. Posttraumatic stress disorder symptoms in resident physicians related to their internship. Acad Psychiatry. 1995;19(3):142-149. doi:10.1007/BF03341425 24442586

