# Supplementary material for: Exposure to Workplace Trauma and Posttraumatic Stress Disorder Among Intern Physicians
Source: JAMA Netw Open. 2021 Jun 8;4(6):e2112837. doi: 10.1001/jamanetworkopen.2021.12837 (PMC8188264; doi:10.1001/jamanetworkopen.2021.12837)
Supplement: Supplement. — eTable 1. Association of Demographic Characteristics of Intern Physicians With Work-Related Trauma Exposure eTable 2. Multivariable Associations of Work-Related, Psychosocial, and Psychological Factors With Work-Related Trauma Exposure eTable 3. Association of Demographic Characteristics of Intern Physicians With Work-Related PTSD eTable 4. Multivariable Associations of Work-Related, Psychosocial, and Psychological Factors With Work-Related PTSD eFigure. Questionnaire Assessing Stressful Life Events Over the Past Three Months [file jamanetwopen-e2112837-s001.pdf]

Supplemental Online Content

Vance MC, Mash HBH, Ursano RJ, et al. Exposure to workplace trauma and posttraumatic stress disorder among intern physicians. *JAMA Netw Open.* 2021;4(6):e2112837. doi:10.1001/jamanetworkopen.2021.12837

eTable 1. Association of Demographic Characteristics of Intern Physicians With Work-Related Trauma Exposure

eTable 2. Multivariable Associations of Work-Related, Psychosocial, and Psychological Factors With Work-Related Trauma Exposure

eTable 3. Association of Demographic Characteristics of Intern Physicians With Work-Related PTSD

eTable 4. Multivariable Associations of Work-Related, Psychosocial, and Psychological Factors With Work-Related PTSD

eFigure. Questionnaire Assessing Stressful Life Events Over the Past Three Months

This supplemental material has been provided by the authors to give readers additional information about their work.

© 2021 Vance MC et al. *JAMA Network Open.*

eTable 1. Association of demographic characteristics of intern physicians with work-related trauma exposure

	Univariable	Multivariable[a]
	**OR (95% CI)**	**OR (95% CI)**
**Demographic characteristics**		
Gender		
Male	1.00 (–)	1.00 (–)
Female	1.04 (0.82-1.32)	1.03 (0.81-1.31)
χ^2_1	0.11	.05
Current Age		
< 25	2.17 (0.70-6.72)	2.05 (0.65-6.45)
26-30	2.44 (0.81-7.34)	2.26 (0.74-6.86)
31-35	2.11 (0.66-6.75)	1.91 (0.59-6.13)
> 35	1.00 (–)	1.00 (–)
χ^2_3	3.21	2.74
Race/Ethnicity		
White	1.37* (1.08-1.74)	1.37* (1.07-1.75)
Non-white	1.00 (–)	1.00 (–)
χ^2_1	6.48*	6.19*
Marital Status		
Not married	1.01 (0.77-1.31)	1.05 (0.80-1.39)
Married	1.00 (–)	1.00 (–)
χ^2_1	0.01	0.14

Note. Multivariable model n = 1127

[a]Multivariable model was adjusted for demographic characteristics (gender, current age, race/ethnicity, and marital status).

*$p < .05$

Legend: OR = odds ratio; CI = confidence interval

© 2021 Vance MC et al. *JAMA Network Open.*

eTable 2. Multivariable associations of work-related, psychosocial, and psychological factors with work-related trauma exposure[a]

	**OR (95% CI)**
**Work-related factors**	
Specialty	
Internal Medicine	1.0 (–)
Surgery	1.58 (0.88–2.83)
Obstetrics/Gynecology	2.07* (1.05–4.09)
Pediatrics	1.25 (0.62–2.53)
Psychiatry	1.38 (0.75–2.56)
Emergency Medicine	1.84 (0.88–3.83)
Medicine/Pediatrics	3.15* (1.58–6.30)
Family Practice	2.90* (1.13–7.44)
Transitional	1.43 (0.77–2.65)
Anesthesiology	1.12 (0.51–2.46)
Other	1.24 (0.64–2.40)
χ^2_{10}	20.94*
Average number of hours worked[b]	1.01* (1.00–1.02)
χ^2_1	7.33*
**Psychosocial factors**	
Early family environment	1.03* (1.02–1.05)
χ^2_1	21.42*
Stressful life experiences (Baseline)[c]	1.70* (1.28–2.25)
χ^2_1	13.54*
Stressful life experiences (Q1 to Q4)[d]	1.16* (1.03–1.33)
χ^2_1	5.45*
Sexual orientation[e]	1.45 (0.95–2.21)
χ^2_1	2.91
**Psychological factors**	
Lifetime history of depression[f]	1.47* (1.16–1.84)
χ^2_1	9.98*
Current depression[g] (Q4)	1.58* (1.17–2.14)

© 2021 Vance MC et al. *JAMA Network Open.*

χ^2_1	8.84*
Current anxiety[h] (Q4)	1.23 (0.89–1.68)
χ^2_1	1.57

Note. n = 1127

[a]Each variable was examined in a separate multivariable model that adjusted for demographics (gender, current age, race/ethnicity, and marital status).

[b]Mean of hours reported (Q1 to Q4).

[c]Cumulative stressful life experiences (0 = no, 1 = yes; Baseline = past 3 months).

[d]Cumulative stressful life experiences (0 = no, 1 = yes; Q1 to Q4).

[e]Sexual orientation: 0 = Heterosexual, 1 = LBGTQ.

[f]Lifetime history of depression reported at baseline (0 = no; 1 = yes).

[g]Current depression: Scoring 10 or higher on the PHQ-9, indicating moderate to severe depression (0 = no, 1 = yes), assessing symptoms over the past 2 weeks.

[h]Current anxiety: Scoring 10 or higher on the GAD-7 (0 = no, 1 = yes), assessing symptoms over the past 2 weeks).

$*p < .05$

Legend: OR = odds ratio; CI = confidence interval; Q = quarter (every 3 months, with Q0 = baseline and Q4 = 12 months); LGBTQ = lesbian, gay, bisexual, transgender, queer/questioning; PHQ-9 = Patient Health Questionnaire-9; GAD-7 = Generalized Anxiety Disorder-7

© 2021 Vance MC et al. *JAMA Network Open.*

eTable 3. Association of demographic characteristics of intern physicians with work-related PTSD[a]

	Univariable	Multivariable[b]
	**OR (95% CI)**	**OR (95% CI)**
**Demographic characteristics**		
Gender		
Male	1.00 (–)	1.00 (–)
Female	1.49 (0.99-2.26)	1.45 (0.95-2.21)
χ^2_1	3.61	3.03
Current Age		
< 25	1.07 (0.11-10.08)	0.77 (0.08-7.43)
26-30	0.91 (0.10-8.27)	0.70 (0.08-6.39)
31-35	1.02 (0.10-10.10)	0.80 (0.08-8.04)
> 35	1.00 (–)	1.00 (–)
χ^2_3	0.37	0.35
Race/Ethnicity		
White	1.40 (0.91-2.14)	1.49 (0.96-2.32)
Non-white	1.00 (–)	1.00 (–)
χ^2_1	2.35	3.22
Marital Status		
Not married	1.51 (0.94-2.42)	1.65* (1.01-2.69)
Married	1.00 (–)	1.00 (–)
χ^2_1	2.92	3.93*

Note. Multivariable model n = 637
[a]Probable PTSD is defined as scoring 3 or greater on the PC-PTSD-5 during Q4.
[b]Multivariable model was adjusted for demographics (gender, current age, race/ethnicity, and marital status).
*$p < .05$

Legend: PTSD = posttraumatic stress disorder; OR = odds ratio; CI = confidence interval; PC-PTSD-5 = Primary Care PTSD Screen for *DSM-5*; Q = quarter (every 3 months, with Q0 = baseline and Q4 = 12 months);

© 2021 Vance MC et al. *JAMA Network Open.*

eTable 4. Multivariable associations of work-related, psychosocial, and psychological factors with work-related PTSD[ab]

	**OR (95% CI)**
**Work-related factors**	
Specialty	
Internal Medicine	1.0 (–)
Surgery	0.40* (0.16–0.97)
Obstetrics/Gynecology	0.22* (0.06–0.78)
Pediatrics	1.29 (0.70–2.38)
Psychiatry	0.27* (0.08–0.93)
Emergency Medicine	0.67 (0.33–1.43)
Medicine/Pediatrics	1.18 (0.42–3.31)
Family Practice	0.98 (0.47–2.06)
Transitional	0.86 (0.27–2.77)
Anesthesiology	0.42 (0.12–1.53)
Other	0.39* (0.17–0.86)
χ^2_{10}	22.38*
Average number of hours worked[c]	1.02 (1.00–1.04)
χ^2_1	3.59
Concern about medical errors[d]	1.36* (1.15–1.60)
χ^2_1	13.44*
**Psychosocial factors**	
Early family environment	1.02 (1.00–1.04)
χ^2_1	3.32
Stressful life experiences (Baseline)[e]	1.24 (0.81–1.92)
χ^2_1	0.93
Stressful life experiences (Q1 to Q4)[f]	1.56* (1.27–1.91)
χ^2_1	18.24*
Sexual orientation[g]	1.41 (0.78–2.57)
χ^2_1	1.29
**Psychological factors**	

© 2021 Vance MC et al. *JAMA Network Open.*

Lifetime history of depression[h]	2.29* (1.50–3.51)
χ^2_1	14.63*
Current depression[i] (Q4)	3.96* (2.58–6.08)
χ^2_1	39.41*
Current anxiety[j] (Q4)	4.41* (2.81–6.92)
χ^2_1	41.46*

Note. $n = 640$

[a]Each variable was examined in a separate multivariable model that adjusted for demographics (gender, current age, race/ethnicity, and marital status).

[b]Probable PTSD is defined as scoring 3 or greater on the PC-PTSD-5 during Q4.

[c]Mean of hours reported (Q1 to Q4).

[d]Cumulative concern about major medical errors (0 = no, 1 = yes; Q1 to Q4).

[e]Cumulative stressful life experiences (0 = no, 1 = yes; Baseline = past 3 months).

[f]Cumulative stressful life experiences (0 = no, 1 = yes; Q1 to Q4).

[g]Sexual orientation: 0 = Heterosexual, 1 = LBGTQ.

[h]Lifetime history of depression reported at baseline.

[i]Current depression: Scoring 10 or higher on the PHQ-9, indicating moderate to severe depression (0=no, 1=yes).

[j]Current anxiety: Scoring 10 or higher on the GAD-7 (0=no, 1=yes).

*$p < .05$

Legend: PTSD = posttraumatic stress disorder; OR = odds ratio; CI = confidence interval; Q = quarter (every 3 months, with Q0 = baseline and Q4 = 12 months); PC-PTSD-5 = Primary Care PTSD Screen for *DSM-5*; LGBTQ = lesbian, gay, bisexual, transgender, queer/questioning; PHQ-9 = Patient Health Questionnaire-9; GAD-7 = Generalized Anxiety Disorder-7

© 2021 Vance MC et al. *JAMA Network Open.*

eFigure. Questionnaire assessing stressful life events over the past three months.

Stressful Life Events

Please indicate if you have experienced any of the following events during the PAST 3 MONTHS (select all that apply).

- ☐ Death of a family member, significant other or close friend
- ☐ You developed a disabling illness or injury lasting a month or more
- ☐ A disabling physical illness or injury started or got worse in a family member, significant other or close friend
- ☐ A relationship with an intimate cohabiting partner ended
- ☐ You were involved in a physically violent relationship
- ☐ You suffered a significant financial loss or loss of property
- ☐ You had problems with debt i.e. having items repossessed, not having enough money to pay household expenses, lacking money for medical expenses or difficulty paying bills
- ☐ You were physically assaulted or attacked
- ☐ You got married
- ☐ You learned that you or your partner were pregnant
- ☐ You had a child

© 2021 Vance MC et al. *JAMA Network Open.*
